# Accelerated growth of pleomorphic adenoma in a pregnant woman undergoing in vitro fertilization: A case report

**DOI:** 10.1097/MD.0000000000043996

**Published:** 2025-08-15

**Authors:** Liu Yang, Wen Li

**Affiliations:** a Department of Otolaryngology-Head and Neck Surgery, West China Hospital, Sichuan University, Chengdu, Sichuan, P. R. China.

**Keywords:** chondrosarcoma, fat degeneration, in vitro fertilization, pleomorphic adenoma, pregnancy

## Abstract

**Rationale:**

Pleomorphic adenoma of the parotid gland usually demonstrates indolent growth; however, its behavior during pregnancy or hormone therapy remains poorly characterized. This case aims to explore the potential impact of in vitro fertilization (IVF) on the rapid tumor progression and its unusual chondrosarcoma-like pathology, raising clinical awareness of possible IVF-associated glandular changes.

**Patient concerns:**

A 37-year-old female undergoing IVF complained of a left parotid mass that increased in size from undetectable to 6 cm (egg-sized) over 2 weeks. She was anxious about the tumor growth rate.

**Diagnoses:**

Imaging showed a parotid tumor measuring 6 cm × 5 cm × 4 cm. Histopathology revealed chondrosarcoma-like appearance with scattered clusters of glandular epithelial cells. Immunohistochemistry confirmed its salivary origin (CK7 and P63 demonstrated positivity in epithelial cells; S-100 demonstrated positivity in chondroid matrix), combined with other pathological performance, malignancy was excluded. Histopathological examination revealed extensive parotid fat degeneration.

**Interventions:**

The patient underwent tumor resection and superficial parotidectomy 2 months after delivery. Hormonal investigations during IVF and perioperative periods were normal except for relatively reduced testosterone.

**Outcomes:**

Postoperative recovery was uneventful. No recurrence or salivary dysfunction occurred during the 4-year follow-up.

**Lessons:**

This case suggests that pleomorphic adenoma might have active stromal growth (rather than cellular increase) on IVF, mimicking chondrosarcoma. Sex hormone levels were not directly connected with tumor growth, indicating other mechanisms may exist. Further studies are warranted to investigate the underlying mechanisms linking IVF to stromal alterations in salivary gland tumors.

## 1. Introduction

Pleomorphic adenoma is the most common pathological type of parotid gland. It accounts for about 80% of all parotid tumors and is characterized by its slow growth pattern. It may be benign over tens of years. Only a few cases may develop to malignancy or be found to be carcinoma ex pleomorphic adenoma. Surgery is the main modality of treatment while there is also some nonsurgical treatment such as transdermal preparation that can slow the growth rate of tumor because of drug-induced infarction of tumor cell.^[1,2]^ Rapid growth of pleomorphic adenoma may imply malignancy or accompanying with pregnancy, the later has been rarely documented in literature. For the convenience of pregnancy, dozens of medicines whose gains and losses are still not quite clear, has been prescribed for in vitro fertilization (IVF) woman^[3]^ Here we report a case of a patient who underwent IVF and experienced rapid growth of pleomorphic adenoma. Its diagnosis was a great challenge as the pathological manifestation was much like that of chondrosarcoma.^[4]^

## 2. Case presentation

**Figure 1.**
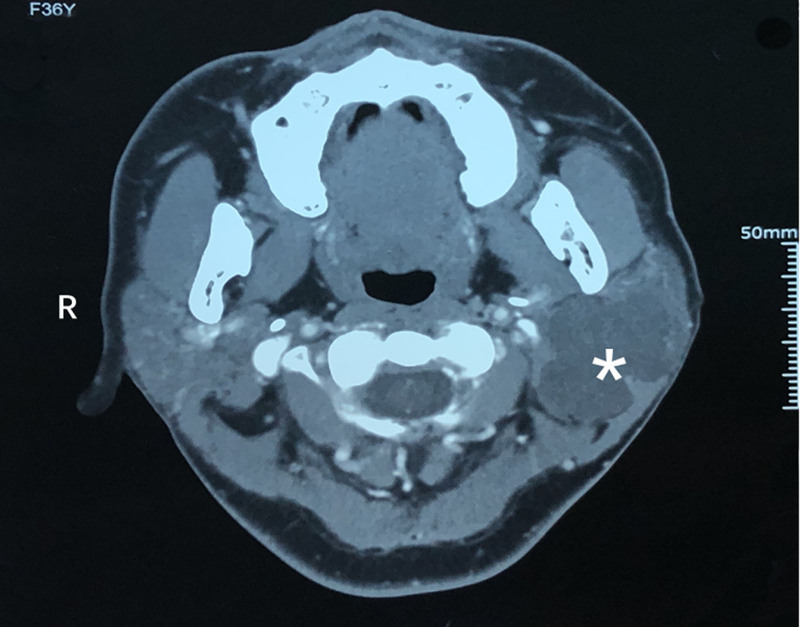
Enhanced CT revealed a nodular lump (*) in the parotid gland on the left side. CT = computed tomography.

**Figure 2.**
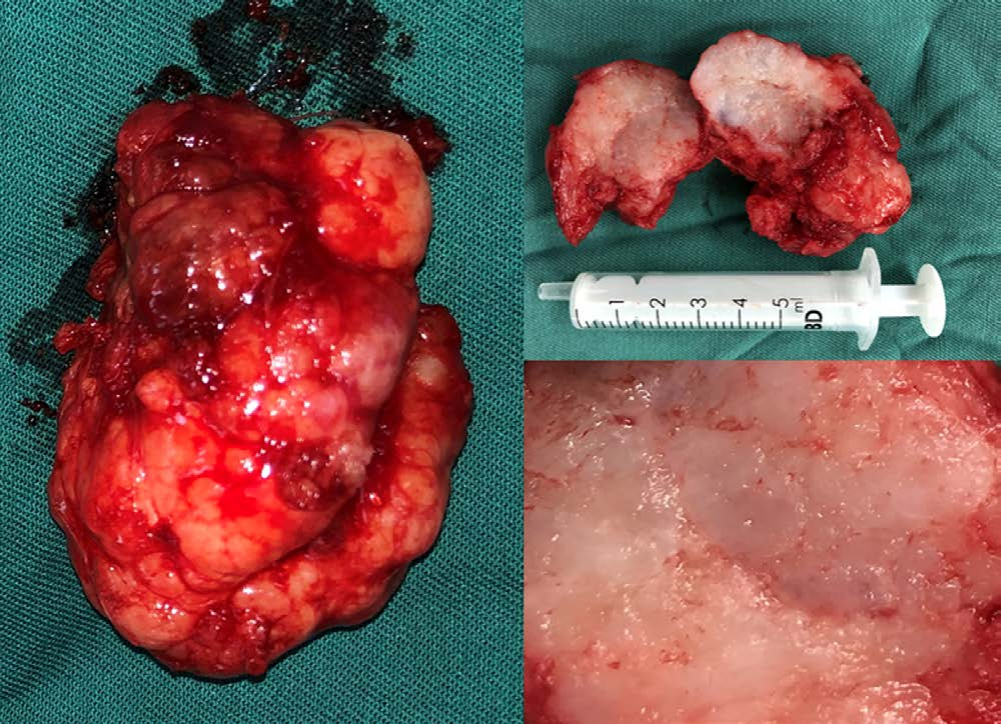
The intact nodular tumor (left), the chondroid cross section (right upper) and stacking of circular crystals appearance on the cross section (right lower).

**Figure 3.**
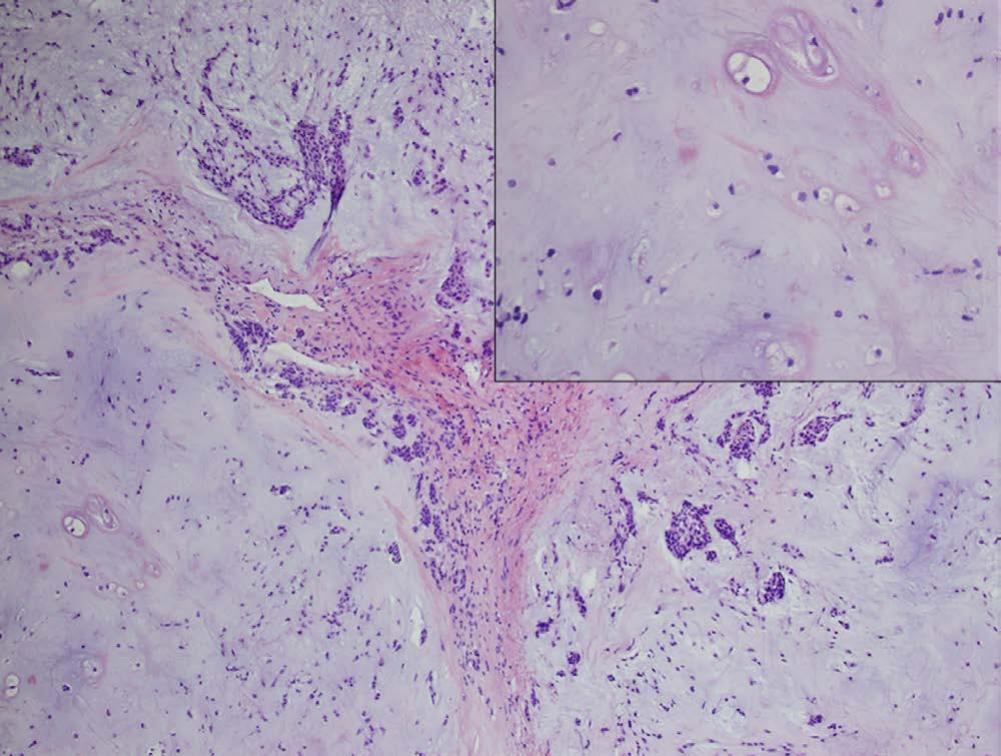
Histopathological study of the tumor revealed the predominant chondrosarcoma-like component and scattered glandular epithelial cell clusters. Inset: magnified view of the lower left area demonstrating grade I chondrosarcoma-like structure, in various differentiated stage from immature chondroblast-like to mature chondrocyte-like cells. (hematoxylin-eosin staining, original magnification × 100).

**Figure 4.**
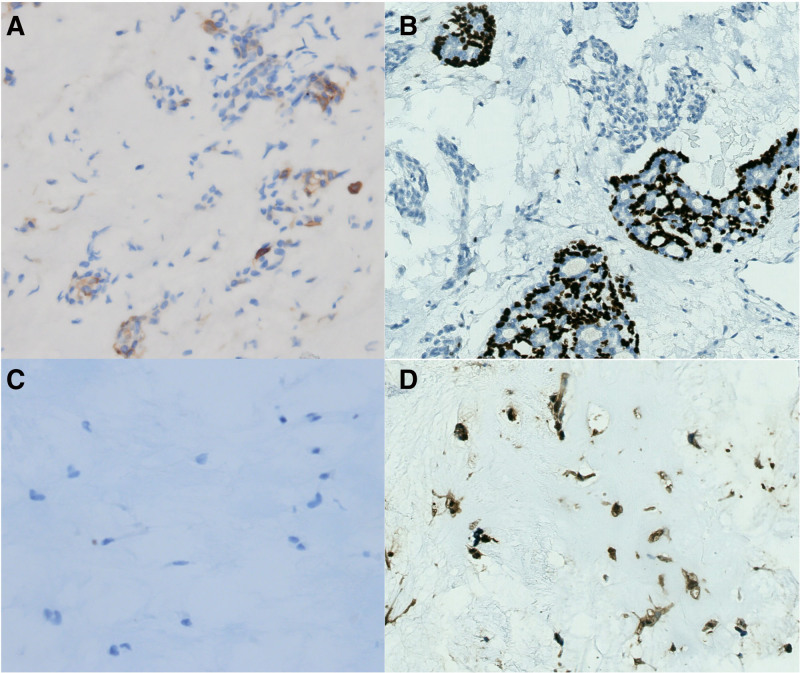
Immunohistochemical study, the glandular epithelial cell clusters revealed positivity for Ck7 and P63; the chondrosarcoma-like component revealed minimal positivity for P63, and strong positivity for S-100. (Immunohistochemistry, [A] CK7, original magnification × 200; [B] P63, original magnification × 200; [C] P63, original magnification × 400; [D] S-100, original magnification × 300).

**Figure 5.**
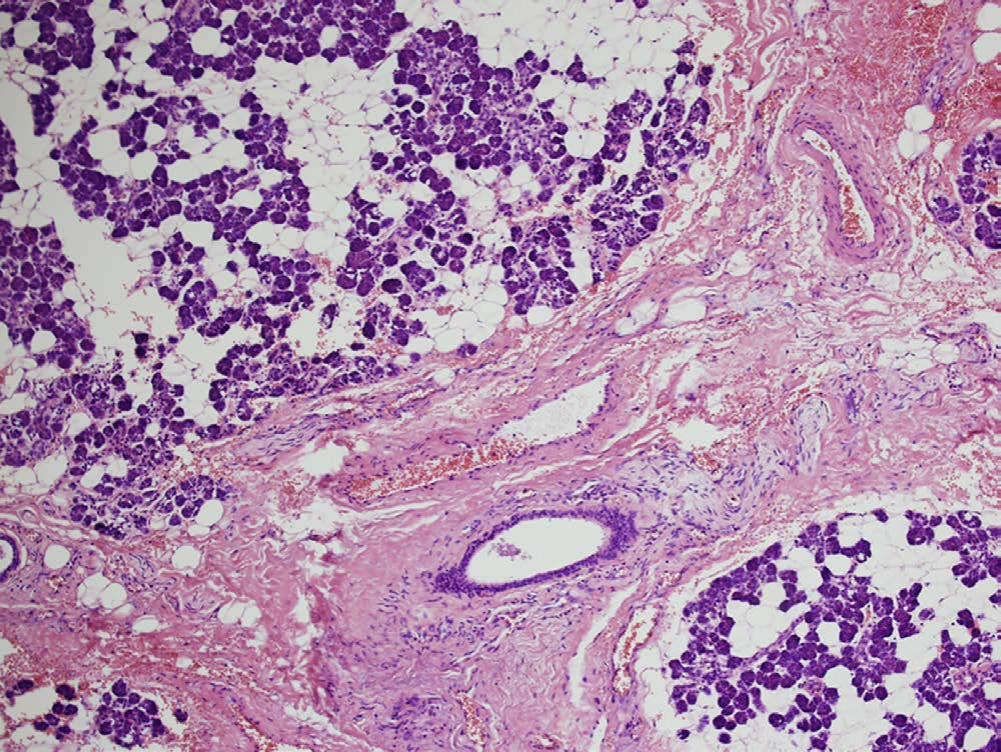
The parotid gland acinar lobules presented with fatty degeneration, interlobular vascular congestion, dilation, and fibrous tissue hyperplasia. (HE staining, original magnification × 100). HE = hematoxylin-eosin.

A 37-year-old female patient was admitted to the hospital on August 27, 2021 due to a lump in the left parotid gland area for 2 months. She was once diagnosed as primary infertility and adopted IVF. she experienced rapid growth of a lump from an unknown size to the size of an egg in 2 weeks. A series of drugs such as Metformin Hydrochloride, Urofollitropin for Injection, Acetic acid Ganiric acid, Prednisone Acetate, Aspirin, Cefaclor, Progynova, Progesterone, Duphaston, Chorionic Gonadotrophin for Injection and Eucommia Ulmoides granules were used from March 19, 2020 to June 23, 2021, as an IVF female usually being prescribed. A survey was conducted on perioperative sex hormones including estradiol, progesterone, human chorionic gonadotropin, luteinizing hormone, testosterone, follicle stimulating hormone and prolactin, all except for testosterone were within normal limits of corresponding physiological phase such as follicular phase or ovulation phase, a slight decrease in testosterone was discovered. She felt overspeed of the tumor and was panic. She had the lump removed 2 months after giving birth. Enhanced computed tomography revealed a lump, about 6 cm × 5 cm × 4 cm in size, located in the left parotid gland (Fig. 1). The patient underwent tumor removal and left superficial parotidectomy during which a white chondroma-like tumor with multiple nodules on the surface and stacking of circular crystals on the cross section, lacking obvious capsule was enucleated (Fig. 2). The gross anatomy of the tumor revealed a homogenously loose chondroma-like tumor, hematoxylin-eosin staining revealed a grade I chondrosarcoma-like picture in various differentiated stage from immature chondroblast-like to mature chondrocyte-like cells, a few clusters of glandular epithelial cell scattered in the chondroid background implied non-chondrogenic origin of the tumor (Fig. 3). Immunohistochemical study revealed the glandular epithelial cell clusters were positive for Ck7 and P63; the chondrosarcoma-like component were minimally positive for P63, and strongly positive for S-100, thus the diagnosis of pleomorphic adenoma was established (Fig. 4). In addition, the parotid gland acinar lobules presented a replacement of adipose tissue which was usually regarded as aging of salivary glands (Fig. 5). The patient recovered uneventfully without facial nerve palsy or wound complications. Periodic imaging at 6-month intervals for the first 2 years and annual ultrasound thereafter showed no evidence of recurrence. The patient reported normal salivary function and no discomfort during the 4-year follow-up, with no clinical signs of xerostomia or parotid swelling. She refused to test the sex hormones and to affirm if fatty degeneration of parotid gland became worse or relieved by ultrasound-guided core needle aspiration biopsy.

## 3. Discussion

Gender-related tumors or tumors with rapid growth during pregnancy can easily be associated with the effects of sex hormones. Sex hormones have specificity for target cells of reproductive organs, in addition, it also has a wide-range impact on cellular metabolism. The role of sex hormone receptors in human cancer development and progression has been well documented in numerous studies, as has the success of sex hormone antagonists in the biological therapy of many human tumors. In salivary gland tumors, little and conflicting information about the role of the sex hormones has been described in most cases and the use of sex hormone antagonists is not contemplated in clinical practic.^[5–8]^ In this case it was discovered that a salivary pleomorphic adenoma may develop in a fast mode in an IVF women as those proved case series in hormone-related breast cancer or prostate cancer, meanwhile the perioperative and IVF period sex hormone assay data suggested no suspicious relation between them. We believe that the duration of the disease is generally reliable because the patient has been consistently receiving facial and neck massages for many years, even during pregnancy, without interruption. Therefore, changes in the size of the facial and neck mass should be easily perceptible. We suppose other underling mechanism other than sex hormones alone may exist for this phenomeno.^[9]^

At first-sight, the gross specimen of tumor and hematoxylin-eosin staining histology of this patient was low-grade chondrosarcoma. Although pleomorphic adenoma of parotid gland is also named as mixed tumor because its components can be osteoid, chondroid or myxoid in form, a mixed tumor with massive chondroid or osteoid component is still uncommon, especially in respect of cellular component rather than gross anatomy or hardness and texture under the knife.^[4,10]^ This means chondroid tissue in the mixed tumor can be generated in the very beginning of tumor genesis, rather than developed in the late stage, this phenomenon has not been explicitly mentioned in literature. It increases the mystery of the tumor genesis of pleomorphic adenomas and expands our knowledge about the various direction during the development of a “mixed tumor.” We don’t know how and when the glandular epithelial cell has turned into chondroid cells. We won’t know if the chondrosarcoma-like cells could develop to mature chondroma at the end because the surgery interrupted the developing process. Immunohistochemical pathological study revealed that the glandular epithelial cell clusters were positive for Ck7 and P63; the chondrosarcoma-like component was minimally positive for P63, and strong positivity for S-100. This may imply the transition from a glandular epithelial cell to a chondroid cell but the deep logic was far from elucidated. We suppose that the chondroid tissue has a rapid growth rate, in addition, from the histopathological study, it can be learned that the rapid growth of tumor size t may be due to an increase of extracellular stroma rather than an increase in the number of tumor cells.

Interestingly, the replacement of parotid gland acinar lobules with adipose tissue, which is also called fatty degeneration, has been noted in the histopathological study. Fatty degeneration is usually regarded as aging of salivary gland, remind us the side-effect of IVF. Because the slight decrease in testosterone, which is regarded as ovarian function decline, will vaguely support the involvement of sex hormones in the aging process of the parotid gland. This has been confirmed in experiments on rats undergoing ovariectomy and become more complicated in human being.^[11–13]^ Advancing age may affect the function of salivary glands. Gradually increasing degrees of acinar atrophy, ductal irregularities, and increase in adipose and fibrovascular tissues are seen in the secretory units of the salivary glands throughout life in healthy individuals, which was also presented in this case (Fig. 5). The former are age-dependent glandular changes and functional impairments, the latter may be IVF-dependent. However, such changes are minor and should not significantly affect the function of salivary glands in comparison with the functional impairment resulted from radiotherapy, immune disease and alcohol intake.^[14–16]^ The results of follow-up of this case also indicated no obvious salivary dysfunction. In this sense, it is encouraging for females in IVF for that IVF has a relatively small impact on salivary gland function, even if we do not have evidence of postpartum reverse recovery from fatty degeneration in patients.

This report is based on a single case, and the inability to longitudinally monitor postoperative hormone profiles or parotid gland recovery limits our understanding of the long-term impact of IVF. Furthermore, the patient declined further imaging or histological confirmation of glandular changes postpartum, restricting the verification of reversibility of fat degeneration.

## 4. Conclusion

This case implies that pleomorphic adenoma of parotid gland may accelerate the growth rate during IVF period, with chondrosarcoma-resembling pathological manifestations. It should be emphasized either from the aspect of growth rate or pathological manifestations in an era of declining birth rates and when IVF is popular. In addition, a study shall be guaranteed to confirm the relationship between IVF practice and the aging tendency of salivary gland.

## Acknowledgments

We thank the patient for granting permission to publish this information.

## Author contributions

**Conceptualization:** Wen Li.

**Project administration:** Wen Li.

**Resources:** Wen Li.

**Supervision:** Wen Li.

**Writing – original draft:** Liu Yang.

**Writing – review & editing:** Wen Li.
